# Effect of the Cornwall Helicopter Ambulance on Ambulance Service Response Time

**Published:** 1992-03

**Authors:** Matthew W. Cooke, Keith M. Porter

**Affiliations:** Senior Registrar, Accident & Emergency Medicine Dudley Road Hospital, Birmingham Medical Officer, West Midlands CARE Team; Consultant Trauma Surgeon Birmingham Accident Hospital Medical Officer, West Midlands CARE Team


### Correspondence

### Effect of the Cornwall Helicopter Ambulance on Ambulance Service Response Time

Sir,

We read the article by Rouse[1] with concern. His study of the Cornwall helicopter relies on one feature of the air ambulance to decide its viability, namely the ORCON standard. The author mentions in his article that it ''lacks credibility for several reasons'' but then sets it as the hallmark for a good service. ORCON only states a response time. This is the second component in the therapy free interval, the first being the time to alerting the emergency services. The Cornwall air ambulance delivers two paramedics to the scene. We are not told if all Cornwall ambulances have one paramedic on board (the other crew member has to drive the land ambulance). The helicopter service may therefore allow more effective deployment of paramedics by increasing their range. It will also allow the patient to be transported to the most appropriate hospital; studies have shown that trauma care is highly variable between hospitals. Land ambulances will usually go to the nearest hospital, which may necessitate futher transfer and therefore a delay in reaching the definitive care facility.

Rouse states that ''the only absolute measurement for evaluating the effectiveness of any therapeutic intervention is a decrease in morbidity and mortality''. Should this therefore not be undertaken before far reaching decisions on the future of this service are made. TRISS methodology is widely recognised as a monitor of effectiveness of trauma care.[2] If this type of study, preferably with comparison with land transfer, were undertaken the information would be more useful than speculation that response time reflects total care quality. One of us (KP) is evaluating the West Midlands Ambulance Service helicopter using TRISS methodology as well as time criteria.

This article discusses the role of the helicopter in primary response but does not inform us of the case mix. We have analysed the 320 missions undertaken in the first nine months of the West Midlands Air Amubulance. Primary responses only account for 69.4% (222 missions) of missions. Secondary response (i.e. calls from an ambulance crew at scene) account for 5.3% (17 missions) and tertiary missions (i.e. inter-hospital transfers) account for 25.3% (81 missions). Therefore, inter-hospital transfer is a major component of the workload.

A recent study[2] has shown an increased survival in intensive care unit patients when transferred by air ambulance as compared to land ambulance. An overall mortality of 20% compared to 38% by road transfer. In the group with a sickness score of over 18 helicopter transfer patients had a 50% survival; it has been previously noted that these patients do not usually survive land transfer.[4] 14 of the patients in this study could not have been transferred by conventional ambulance because of inadequate equipment, power or medical gases.

Improved survival with helicopter transfer may be due to the ability to monitor and treat the patient more aggressively. One helicopter can be equipped with a large amount of expensive equipment and serve several ambulance services. A similarly equipped land ambulance could only serve a small geographic area. Alternatively the survival may be increased due to the lower head to toe forces of a helicopter compared to a land ambulance,[5] as accelerational forces in this direction can cause a marked sympathetic response.[6] Kee et al[3] failed to see the sympathetic response to movement during helicopter transfer that had been previously reported during land transfer.[4]

Like Rouse we would like to suggest two vignettes that highlight the function of the air ambulance.

**Vignette one**
A patient in a remote area away from public roads has a prolonged period before a basic trained ambulance crew can reach him. He is carried across rough ground and then transported to the local hospital where treatment is commenced. He is later transferred to the regional neurosurgical unit. Alternatively two helicopter paramedics could commence treatment on scene and then rapidly transfer him, with full monitoring, to a large Accident & Emergency department in a hospital containing neurosurgical facilities.

**Vignette two**
A young man in terminal cardiac failure requiring intensive monitoring and positive pressure ventilation requires transfer to a supra-regional cardiac transplant unit. The journey by road would take 3 hours including city centre traffic and winding country lanes.

The study by Rouse may show that ORCON standards in a region are not improved by the provision of an air ambulance. The study is useful in enumerating ways of improving ORCON statistics but not necessarily patient care. There is insufficient information to determine the impact of the air amublance on patient outcome. We believe that this information should be obtained before decisions are made on the future deployment of medical helicopter services.

Matthew W. Cooke
Senior Registrar, Accident & Emergency Medicine
Dudley Road Hospital, Birmingham
Medical Officer, West Midlands CARE Team

Keith M. Porter
Consultant Trauma Surgeon
Birmingham Accident Hospital
Medical Officer, West Midlands CARE Team
March 3, 1992

### REFERENCES

1. Rouse A. Effect of the Cornwall Helicopter Ambulance on Ambulance Service Emergency Response Time. *West of England Medical Journal.* 1991: **106(iii):** 68-72.
2. Boyd. C.R., Tolson M.A. & Copes W.S. 1987. Evaluating Trauma Care: thec TRISS method. *The Journal of Trauma:* **27:** 370-8.
3. Kee S.S. Ramage C.M.H., Mendel P. Bristow A.S.E. Interhospital transfers by helicopter: the first 50 patients of the Careflight project. *Journal of the Royal Society of Medicine.* 1992: **85:** 29-31.
4. Bion J.F., Edlin S.A., Ramsey G., McCabe S. Ledingham IMcA. Validation of a prognostic score in the critically ill patients undergoing transport. *BMJ.* 1985: **291:** 432-4.
5. Silbergleit R., Dedrick D.K., Pape J., Burney R.E. Forces acting during air and ground transport on patients stabilised by standard immobilisation techniques. *Annals of Emergency Medicine.* 1991: **20(8):** 875-7.
6. Lauritzen J.B., Lendorf A., Vesterhauge S., Johansen T.S., Heart Rate Response to moderate linear body acceleration: clinical implications in aeromedical evacuation. *Aviation Space & Environmental Medicine.* 1987: **58(3):** 248-51.

